# Socioeconomic position and self-harm among adolescents: a population-based cohort study in Stockholm, Sweden

**DOI:** 10.1186/s13034-017-0184-1

**Published:** 2017-09-06

**Authors:** Bereket T. Lodebo, Jette Möller, Jan-Olov Larsson, Karin Engström

**Affiliations:** 1 Department of Public Health Sciences, Karolinska Institutet, Tomtebodavägen 18a, 17177 Stockholm, Sweden; 2 Department of Women’s and Children’s Health, Karolinska Institutet, 17177 Stockholm, Sweden

**Keywords:** Self-injurious behavior, Adolescent, Social class, Cohort, Sweden

## Abstract

**Background:**

Understanding the association between parental socioeconomic position and self-harm in adolescence is crucial due to its substantial magnitude and associated inequality. Most previous studies have been either of cross-sectional nature or based solely on self-reports or hospital treated self-harm. The aim of this study is to determine the association between parental socioeconomic position and self-harm among adolescents with a specific focus on gender and severity of self-harm.

**Methods:**

A total of 165,932 adolescents born 1988–1994 who lived in Stockholm at the age of 13 were followed in registers until they turned 18. Self-harm was defined as first time self-harm and severity of self-harm was defined as hospitalized or not. Socioeconomic position was defined by parental education and household income. Cox proportional hazards regression were used to estimate hazard ratios (HR) with 95% confidence intervals (CI).

**Results:**

Analyses showed an association between parental socioeconomic position and self-harm. Among adolescents with parents with primary and secondary education compared to tertiary parental education the HR were 1.10 (95% CI 0.97–1.24) and 1.16 (95% CI 1.08–1.25) respectively. Compared to the highest income category, adolescents from the lower income categories were 1.08 (95% CI 0.97–1.22) to 1.19 (95% CI 1.07–1.33) times more likely to self-harm. In gender-stratified analyses, an association was found only among girls. Further, restriction to severe cases eliminated the association.

**Conclusions:**

This study suggested that low parental socioeconomic position is associated with self-harm in adolescence, predominantly among girls. The desertion of an association among severe cases may be explained by differences in suicidal intent and underlying psychiatric diagnosis. Efforts to prevent self-harm should consider children with low parental socioeconomic position as a potential target group.

## Background

Self-harm refers to a range of behaviors in which individuals deliberately initiate actions with an intention to harm themselves regardless of types of motivation or the extent of suicidal intent [1, 2]. This definition is often used because suicidal intent can be problematic to judge as it may be surrounded by ambivalence or even disguise [3]. There is no formal autonomous diagnosis for self-harm without suicidal attempt in ICD 10, DS M-IV or DSM-5. In DSM-5, it has however been included in a section for conditions on which future research is encouraged [4]. Although international variation exists, findings around the world indicate that the prevalence rate of lifetime self-harm in adolescents range between 6 and 18% [5–10]. In Sweden, based on a single item question assessment tool, the prevalence of deliberate self-harm was estimated to 17% [11]. Self-harm has a repetitive nature [12] and it has been shown that the risk of suicide among self-harming individuals is much higher than in the general population [13]. Self-harm is more common among adolescent girls than boys [14–16] and there is also gender differences in the methods of self-harm [17].

Due to the magnitude and gender difference associated with self-harm among adolescents, it is of great importance to further understand the mechanisms of self-harming behavior. The existing literature show that many different factors such as adverse childhood effects [18, 19], bullying [20, 21], neurobiological factors [22, 23] and other social factors [24] are associated with self-harm. Previous studies have also pointed out the impact of socioeconomic factors on self-harm among adolescents and young adults, and this holds irrespective of the measure of socioeconomic position used. A study from UK showed that lower socioeconomic status during childhood is associated with a higher risk of self-harm with suicidal intent among adolescents [25]. A survey from Belgium showed children with unemployed parents and who have low educational level were found be at a higher risk of non-suicidal self-injury (NSSI) [26]. In a cross-sectional study of Swedish adolescents, an inverse relationship has been found between parental socioeconomic status and intentional injury risk among adolescents admitted to hospitals for self-inflicted injury [27]. In a recent Swedish national study, socioeconomic factors explained the higher risk of hospitalization for self-inflicted injury among youth in ethnic minorities [28]. In previous studies, not much attention has been paid to potential gender differences in the association between socioeconomic position and self-harm.

The majority of available studies regarding the association between socioeconomic position (SEP) and self-harm have been cross-sectional in design and based on either solely diagnoses of self-harm in inpatient care or on self-reports of non-clinical self-harming behaviors. Self-harm treated in outpatient care has not been studied much yet. In this longitudinal study, we exploit Sweden’s extensive and high-quality registers for both inpatients and outpatient cases of self-harm based on a large population of adolescents in Stockholm. The overall aim of this study is to determine the association between parental socioeconomic position and risk of self-harm among adolescents with a specific emphasis on gender difference and severity of self-harm.

## Methods

This cohort study was based on the Stockholm Youth Cohort (SYC), a record-linkage comprising all children aged 0–17 years who lived in Stockholm County at any time from 2001 to 2011. Data in SYC is derived from national and regional administrative and health care registers. Adolescents in SYC were identified through the total population register [29] and linked to their parents using the multi-generation register [30]. Parent(s) in this study refer to the adult(s) with whom the adolescent was registered as living with, which includes biological, adoptive and ‘other’ parent (e.g. a foster parent). Adolescents who had ‘other’ parent as a second parent were considered to have only one parent since it is only possible to determine the ‘other’ parent if he/she lives in the same one-family house, but not if he/she lives in an apartment house. A person can only be registered in one address even though some children live part-time in two families.

### Study population

The study population consisted of 169,262 adolescents comprising of seven birth cohorts, born between 1988 and 1994, who lived in Stockholm County at the age of 13, withdrawn from SYC. The study period extended from 2001 to 2011, with each of the seven birth cohorts being followed for 5 years, from age 13 to 17. Adolescents with missing values on at least one of the explanatory variables or the outcome variable (n = 3300) were excluded and the final study population consisted of 165,932 adolescents.

### Self-harm

First-time self-harm, from here-on referred to as self-harm, was the main outcome of the study and was ascertained through individual record linkage to national administrative registers and regional health care registers, covering all pathways of diagnosis and care related to self-harm, except private clinics. The registers were: (1) the VAL database, a Stockholm County register on public health care services which includes out-patient, in-patient and primary care, (2) the Cause of Death register and (3) Pastill, a clinical database covering all visits to child and adolescent psychiatry in Stockholm. Self-harm was defined according to the tenth revision of the World Health Organization (WHO) Classification of Diseases (ICD-10) (Intentional self-harm X60–X84) in the VAL database, Cause of Death register and Pastill. In Pastill, self-harm was additionally defined by a diagnosis of suicidal attempt and by self-harm as a contact reason. Only the first episode of self-harm during age 13–17 was used.

Severity of self-harm was defined based on the level of care rendered to individuals: those who received inpatient care for self-harm were considered as severe cases and those who received outpatient care for self-harm were considered as less severe cases. The most common reasons to be hospitalized for self-harm in Stockholm County is suspected or identified suicidal attempt. It is also more common among those hospitalized to have substance related disorders and, to some extent, anxiety disorders as underlying psychiatric diagnoses, whereas psychosis and bipolar disorders, neurodevelopment disorders as well as disruptive, impulse-control and conduct disorders were less common in both groups. Depressive disorders and anxiety disorders are the most common comorbid psychiatric diagnoses in self-harm both with and without hospitalization. Hospitalization requiring admission for at least one night was considered as inpatient care.

### Socioeconomic position

Socioeconomic position (SEP), the main exposure, was measured the year the adolescent turned 12. SEP was measured in two ways, parental education and household disposable income. Information on SEP was extracted from the longitudinal integration database for health insurance and labor market studies (LISA). Level of education was categorized into three categories based on number of years of completed education: up to 9 years (primary education), 10–12 years (secondary education) and >12 years (tertiary education). The highest educational achievement of either parent was used to define parental education. Household disposable income was categorized into quintiles, with consideration of year of income determination in addition to the actual income to ensure that approximately equivalent income groups were compared over time. The first and fifth quintiles represented the lowest and highest household income categories respectively.

### Covariates

Demographic factors—age, gender and parental country of birth—were assessed using information from the Total population register. Age was used as a continuous variable. Parental country of birth was categorized in three groups: Sweden if a single parent or both parents were born in Sweden, outside Sweden if a single parent or both parents were born outside of Sweden, and mixed if one parent was born in Sweden and the other outside Sweden.

Social and economic factors used in this study were number of parents in the household and receipt of welfare benefit. A household was regarded as having received welfare benefit if anyone in the household received benefit, once or several times, during the year the adolescent turned 12; the data was extracted from LISA. History of mental disorder of biological parent was defined when a biological parent was hospitalized for at least one night due to any mental disorder. The information was obtained from the National Hospital Discharge register from 1964 until the adolescent turns 13 years old.

### Statistical analysis

The characteristics of the cohort were described using descriptive statistics. Incidence rates for self-harm were calculated per 100,000 person-years. Proportionality of the hazard assumption was checked using log minus log graph. Analyses were performed using Cox proportional hazard regression to assess the association between self-harm, SEP and other relevant covariates and to estimate hazard ratios (HR) with corresponding 95% confidence intervals (CIs). Time under risk was calculated using the entry date defined as the date the adolescent turned 13 years of age, and the exit date as the date of the first-time diagnosis of self-harm, date of death of any cause, date of moving out of Stockholm County or the end of follow-up, whichever came first.

Stratified analyses were performed by severity of self-harm, to assess the role of severity of the self-harm; and by gender to address gender differences. We considered receipt of welfare benefits, parental country of birth, number of parents in the household and mental disorder of biological parent as potential confounders/mediators. SAS version 9.3 was used for all statistical analyses.

## Results

A summary of the characteristics of the cohort is presented in Table 1. The total sample size was 165,932 (51.3% boys and 48.7% girls).

**Table 1:** Characteristics of the cohort and cases of first-time self-harm (N = 165,932)

Characteristic	Distribution of the cohort	Incidence of first-time self-harm per 100,000 person-years		
	N (%)	All	Boys	Girls
Total	165,932 (100)	400	–	–
Gender				
Boys	85,182 (51.3)	143	–	–
Girls	80,750 (48.7)	675	–	–
Parental education				
Primary	15,829 (9.5)	469	159	796
Secondary	69,564 (41.9)	453	154	773
Tertiary	80,539 (48.6)	341	131	567
Household income				
1st quintile (Lowest)	32,659 (19.7)	381	123	658
2nd quintile	33,239 (20.0)	459	145	795
3rd quintile	33,356 (20.1)	442	176	727
4th quintile	33,344 (20.1)	383	157	628
5th quintile (highest)	33,334 (20.1)	335	116	567
Receipt of welfare				
No	15,606 (93.8)	394	142	664
Yes	10,326 (6.2)	486	166	837
Parental country of birth				
Sweden	107,470 (64.8)	400	140	676
Mixed	21,790 (13.1)	505	184	852
Outside Sweden	36,672 (22.1)	339	130	565
Number of parents in the household				
One	49,256 (29.7)	567	207	951
Two	116,676 (70.3)	331	117	559
History of mental disorder of biological parent				
No	148,718 (89.6)	365	132	615
Yes	17,214 (10.4)	705	244	1206

A total of 3230 adolescents had a documentation of self-harm during the study period, which correspond to an incidence rate of 400 per 100,000 person-years, substantially higher for girls than boys. The incidence rate of self-harm was highest among adolescents whose parents had primary education and lowest among adolescents whose parents had tertiary education. The incidence rate of self-harm was highest among adolescents from households with 2nd quintile income category and lowest among adolescents from households with 5th income quintile category.

**Fig. 1:**
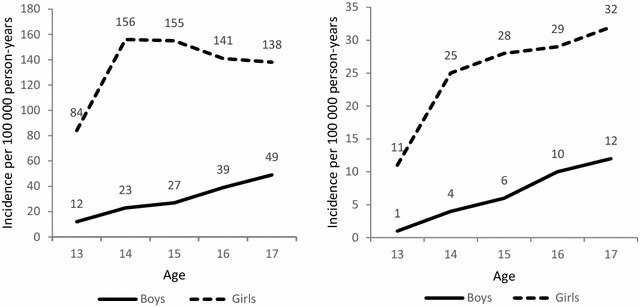
**a** Gender difference in the incidence rate per 100,000 person-years of first-time self-harm. **b** Gender differences in the incidence rate per 100,000 person-years of first-time severe self-harm

First-time self-harm among boys was most common at age 17 and least common at age 13. Among girls, first-time self-harm was most common at age 14 and least common at age 13 (Fig. 1a). About 16% (n = 516) of those with first-time self-harm were admitted to a hospital for care. Among those, the proportion of girls was almost three-times higher than boys (75.8% vs 24.9%) (Fig. 1b). The mean age of first-time self-harm in this cohort was 15.7 (SD = 1.3) (not shown).

Table 2 shows HRs of self-harm for ‘all’ and ‘severe cases’. In the partially adjusted model, all categories of parental education and household income compared to the reference groups remained associated with higher risk of self-harm among adolescents. In the fully adjusted model, secondary parental education compared to tertiary parental education was associated with higher risk of self-harm among adolescents. Though CI included one, the risk of self-harm was higher among adolescents with parents with primary education (Model 3). In the fully adjusted model, the risk of self-harm was higher among adolescents with parents from lower household income categories when compared to the 5th quintile income category, though CI included one for the 4th quintile income category (Model 3). In analyses limited to inpatient cases of self-harm, no association was found for both parental education and household income in the adjusted models (Model 3). Less severe cases showed similar results to those of all cases (numbers not shown).

**Table 2:** Hazard ratios (HR) with 95% confidence intervals (CI) of adolescent first-time self-harm by parental education and household income

	All cases			Severe cases		
	Model 1 HR (95% CI)	Model 2 HR (95% CI)	Model 3 HR (95% CI)	Model 1 HR (95% CI)	Model 2 HR (95% CI)	Model 3 HR (95% CI)
Parental education						
Primary	1.37 (1.22–1.55)	1.37 (1.21–1.54)	1.12 (0.99–1.24)	1.39 (1.07–1.82)	1.24 (0.94–1.63)	1.23 (0.92–1.64)
Secondary	1.33 (1.23–1.43)	1.29 (1.20–1.39)	1.18 (1.09–1.27)	1.13 (0.94–1.36)	1.09 (0.90–1.31)	1.08 (0.89–1.30)
Tertiary	1.00 (REF)	1.00 (REF)	1.00 (REF)	1.00 (REF)	1.00 (REF)	1.00 (REF)
Household income						
1st quintile	1.15 (1.02–1.29)	1.20 (1.07–1.36)	1.13 (1.00–1.27)	1.04 (0.78–1.38)	0.96 (0.71–1.29)	0.97 (0.72–1.31)
2nd quintile	1.37 (1.22–1.53)	1.34 (1.20–1.50)	1.20 (1.07–1.34)	1.08 (0.82–1.41)	1.00 (0.76–1.32)	0.99 (0.75–1.30)
3rd quintile	1.32 (1.18–1.47)	1.30 (1.16–1.45)	1.18 (1.05–1.32)	1.01 (0.77–1.34)	0.99 (0.75–1.32)	0.98 (0.74–1.30)
4th quintile	1.15 (1.03–1.29)	1.14 (1.01–1.27)	1.07 (0.96–1.21)	0.90 (0.67–1.21)	0.87 (0.65–1.18)	0.86 (0.64–1.16)
5th quintile	1.00 (REF)	1.00 (REF)	1.00 (REF)	1.00 (REF)	1.00 (REF)	1.00 (REF)

Model 1: adjusted for gender

Model 2: adjusted for gender, parental country of birth and history of mental disorder of biological parent

Model 3: adjusted for gender, parental country of birth, history of mental disorder of biological parent, receipt of welfare and number of parents in the household

Table 3 presents HRs of gender-stratified analyses between parental SEP and risk of self-harm. Among boys, parental education was not found to be associated with self-harm. Though the point estimates were higher in most of the categories, the only association found between household income and self-harm was for the third and fourth quintile income categories in the crude and partially adjusted model which for the fourth quintile was eliminated after full adjustment.

**Table 3:** Gender stratified hazard ratios (HR) with 95% confidence intervals (CI) of adolescent first-time self-harm by parental education and household income

	Boys			Girls		
	Model 1 HR (95% CI)	Model 2 HR (95% CI)	Model 3 HR (95% CI)	Model 1 HR (95% CI)	Model 2 HR (95% CI)	Model 3 HR (95% CI)
Parental education						
Primary	1.21 (0.92–1.60)	1.17 (0.88–1.56)	0.95 (0.71–1.28)	1.41 (1.24–1.60)	1.42 (1.24–1.62)	1.16 (1.02–1.32)
Secondary	1.18 (0.99–1.39)	1.14 (0.96–1.35)	1.03 (0.86–1.22)	1.36 (1.26–1.48)	1.33 (1.23–1.44)	1.22 (1.12–1.32)
Tertiary	1.00 (REF)	1.00 (REF)	1.00 (REF)	1.00 (REF)	1.00 (REF)	1.00 (REF)
Household income						
1st quintile	1.09 (0.83–1.43)	1.07 (0.80–1.41)	1.02 (0.76–1.36)	1.15 (1.02–1.31)	1.23 (1.08–1.41)	1.15 (1.01–1.32)
2nd quintile	1.25 (0.96–1.63)	1.20 (0.92–1.57)	1.07 (0.81–1.40)	1.39 (1.23–1.57)	1.37 (1.22–1.55)	1.23 (1.09–1.39)
3rd quintile	*1.52 (1.18–1.96)*	*1.48 (1.14–1.91)*	*1.33 (1.03–1.73)*	1.27 (1.12–1.44)	1.26 (1.11–1.42)	1.15 (1.01–1.30)
4th quintile	*1.37 (1.05–1.77)*	*1.34 (1.03–1.74)*	1.26 (0.97–1.64)	1.10 (0.97–1.25)	1.09 (0.96–1.24)	1.03 (0.91–1.17)
5th quintile	1.00 (REF)	1.00 (REF)	1.00 (REF)	1.00 (REF)	1.00 (REF)	1.00 (REF)

Model 1: crude

Model 2: adjusted for parental country of birth and history of mental disorder of biological parent

Model 3: adjusted for parental country of birth, history of mental disorder of biological parent, receipt of welfare and number of parents in the household

In contrast, among girls, parental education was associated with self-harm in both crude and adjusted models. After full adjustment, girls with primary parental education were 1.16 times more likely to develop self-harm than those whose parental education was tertiary education. Girls with secondary parental education were 1.22 times more likely to develop self-harm compared to those girls with tertiary parental education. Household income was associated with self-harm among girls except for the fourth quintile income category in all the models. When compared to the fifth quintile income category, girls from other categories were 1.03–1.23 times more likely to develop self-harm (Table 3, Model 3).

HRs of gender-stratified analyses between parental SEP and risk of severe cases of self-harm are presented in Table 4. Neither parental education nor household income showed association with severe cases of self-harm among both boys and girls in the adjusted models (Model 3).

**Table 4:** Gender stratified hazard ratios (HR) with 95% confidence intervals (CI) of adolescent first-time severe self-harm by parental education and household income

	Boys			Girls		
	Model 1 HR (95% CI)	Model 2 HR (95% CI)	Model 3 HR (95% CI)	Model 1 HR (95% CI)	Model 2 HR (95% CI)	Model 3 HR (95% CI)
Parental education						
Primary	1.02 (0.56–1.86)	0.84 (0.46–1.56)	0.82 (0.43–1.56)	1.41 (1.07–1.85)	1.20 (0.90–1.60)	1.18 (0.87–1.59)
Secondary	1.37 (0.96–1.95)	1.34 (0.94–1.91)	1.28 (0.89–1.84)	1.09 (0.90–1.32)	1.03 (0.84–1.25)	1.02 (0.83–1.24)
Tertiary	1.00 (REF)	1.00 (REF)	1.00 (REF)	1.00 (REF)	1.00 (REF)	1.00 (REF)
Household income						
1st quintile	0.82 (0.47–1.45)	0.76 (0.41–1.40)	0.91 (0.49–1.68)	1.18 (0.88–1.58)	1.02 (0.75–1.38)	1.01 (0.74–1.38)
2nd quintile	1.08 (0.64–1.82)	0.90 (0.52–1.54)	0.90 (0.52–1.55)	1.09 (0.82–1.45)	0.98 (0.73–1.30)	0.95 (0.71–1.28)
3rd quintile	1.00 (0.60–1.66)	1.01 (0.61–1.69)	0.94 (0.56–1.58)	1.00 (0.74–1.35)	0.97 (0.72–1.31)	0.96 (0.71–1.30)
4th quintile	0.84 (0.48–1.47)	0.80 (0.46–1.40)	0.72 (0.41–1.27)	0.97 (0.72–1.33)	0.92 (0.68–1.26)	0.91 (0.67–1.25)
5th quintile	1.00 (REF)	1.00 (REF)	1.00 (REF)	1.00 (REF)	1.00 (REF)	1.00 (REF)

Model 1: crude

Model 2: adjusted for parental country of birth and history of mental disorder of biological parent

Model 3: adjusted for parental country of birth, history of mental disorder of biological parent, receipt of welfare and number of parents in the household

## Discussion

This study suggests that, though the magnitude of the effect is not large, low parental SEP is associated with increased risk of self-harm among adolescents, predominantly among girls. It also indicates that this association is not present for adolescents with more severe self-harm.

The association between parental SEP and risk of self-harm among adolescents indicated in this study is consistent with previous findings [25, 26, 31–35]. Both household income and parental education were inversely associated with a risk of self-harm. The effect of household income was seen in most income categories with a stronger effect for the lower three income categories. Findings from a UK birth cohort showed a linear association between decreasing household income and self-harm [35]. Other studies from Belgium and Australia revealed an inverse association between family income and NSSI [25, 26]. Previous studies have also shown an association between lower parental and/or maternal education and increased risk of self-harm among adolescents [26, 33, 34]. No association was found for primary education category in this study, which could be explained by a lower healthcare utilization in this group of people. More than 50% of parents with primary education were born outside Sweden, a factor that was related to lower utilization.

The result of this study, suggesting SEP is inversely associated with the risk of self-harm among adolescents, is in accordance with the social causation theory which states that encountering socioeconomic hardship augments the risk of subsequent mental illness [36]. The excess risk of self-harm attributed to SEP can be explained by several mechanisms. First, adolescents raised in unfavorable circumstances in socially deprived families are prone to multiple stressors, increasing their predisposition to mental health disorders [37]. Second, lower SEP may be linked with a varied array of undesirable consequences for parents, such as substance abuse and mental and/or physical illness [38], which may influence the quality of parenting [39]. A third underlying mechanism may be social exclusion created by an absence of family assets, which may result in lowered self-esteem and feelings of seclusion as well as depressive symptoms during adolescence [40], which in turn are recognized causes of self-harm [41].

The magnitude of the effect found in the associations, after adjustment for demographic, social and economic factors, is rather low. This was mainly evident after adjusting for receipt of welfare benefit and number of parents in the household. These factors could also play a role as mediators in the association between SEP on self-harm. Adjusting for mediators could lead to over-adjustment which would cause an underestimation of the effect.

Supporting some prior evidence [27] and contradicting some [34, 42], this study pointed out that the association between parental SEP and risk of self-harm was eliminated when the analyses were restricted to severe cases of self-harm after controlling for demographic and other social and economic factors. Elimination of the observed association between parental SEP and risk of self-harm for inpatient cases may indicate that differences in health care utilization are less pronounced if adolescents experience a more severe episode of self-harm mandating hospitalization. In Sweden, lower socioeconomic groups refrain to a larger extent from seeking medical care they need [43, 44] and increment in these trends has been observed [45]. However, since suicidal intent is more common among those being hospitalized, as is substance-related disorders, fewer in this group may avoid seeking care because of economic or cultural reasons.

The impact of parental SEP on the risk of self-harm seem to differ by gender. Low parental SEP was associated with higher risk of self-harm among girls only. This result was in accordance with a study from the US which examined the sex differences in the effect of parental education on subsequent mental health problem and indicated that females are more affected [46]. A recent study from Japan reported that among women, unlike men, parental education was associated to major depression [47]. In contrast many studies have not found significant gender difference in the association [48–50]. Boys and girls may react differently to environmental circumstances and differ in their stress response, then making parental SEP more important for self-harm behavior to one gender than the other [51]. In social relations, a tendency has been noticed for girls to exhibit a strong affiliative style, referring to an inclination for tight emotional connection, closeness and receptiveness within interpersonal relations [52]. In the view of this, socioeconomic hardships could trigger a more a pronounced adverse effect on the mental health of girls than boys. It is also possible that childhood adversities affect boys in a different way [52], including alcohol abuse and antisocial personality, which is not captured by self-harm in this study [53]. An alternative explanation is that despite the population based design and large study sample, the effect among boys could not be determined as statistically significant due to small number of cases.

### Strengths and limitations

This population-based study with a large cohort of adolescents yielded high power with long follow-up time and full coverage of events of self-harm from almost all pathways of diagnoses and care to self-harm in Stockholm County. Since the health care system as well as the composition of the population is similar between the big cities of Sweden, the results of the study can be generalized to the population of those big cities and other populations within a similar context. We believe that we eluded some of the limitations confronted by previous studies—specifically, recall bias and loss to follow-up which could have led to selection bias. The longitudinal nature of the study gave us an opportunity to make conclusions about causality. Inclusion of non-hospitalized (less severe) cases of self-harm in this study helps to address this rarely studied portion of the self-harming population and to make more comprehensive conclusions. The gap in the data caused by missing information about the parental education, household income and other covariates were few ranging between 1.3 and 2.0% (n = 3300). And there was no significant difference found in risks of self-harm because of these missing values. Using multiple variables to assess SEP, which measures different aspects of the concept, helped to give a broader perspective as underlying pathways are multifaceted and complex. Literature suggested that variables which measure SEP should not be used interchangeably as they measure different aspects of socioeconomic positions and refer into different causal mechanisms [54, 55].

One limitation in this study lies in the use of health care registers and limits our analyses to cases of self-harm for which care has been sought. Compared to other recent population-based survey studies, the figures for self-harm are lower in this study which indicate that many adolescents who self-harm do not seek treatment [56]. The tendency to seek care may differ depending on method used, which could explain part of the differences between boys and girls. High priority is given to equity in health in Sweden [57] and the target of the Swedish Health Care Act is equity in opportunity to use healthcare depending on need [58]. However, studies show that health-care utilization is not always strictly linked to health status and need, several factors can impact whether ill-health status leads into utilization of healthcare [57], and several studies have revealed disproportionately lower utilization of healthcare services by people with low SES and ethnic minorities [59, 60]. In Sweden, lower socioeconomic groups refrain to a larger extent from seeking medical care they need [43, 44] and increment in these trends has been observed [45]. Though this is a somewhat lesser problem with regard to children, since most medical services are free for children [61], lack of time may also play a role. Hence, the increased risk found among adolescents with low SEP is likely an underestimation. On the other hand, parents of adolescents with higher SEP may choose to visit private psychiatric clinics, whose data was not included in this analysis, which would lead to a slight overestimation of our results. It is important to examine whether the degree of underreporting is comparable across SEP categories.

Another concern in this study was a possible non-differential misclassification of parental SEP and other social characteristics which could have occurred due to two reasons. First, only one household was recognizable for adolescents who passed equivalent or different amount of time residing in the homes of separated parents, as children in Sweden are registered at a single address [62]. Second, it was not possible to determine a second parent if he or she was not biological or adoptive parent, as the information on the second parent when non-biological/adoptive was differential due to housing conditions, and housing conditions are related to one’s socioeconomic position. Both by recognizing only one of two households and by excluding the second parent when non-biological/adoptive, some adolescents may have been classified to a lower SEP than they should. Such misclassifications would lead to underestimation of the effect.

## Implications

The association between parental SEP and self-harm among adolescents suggests that prevention strategies should apply the principle of proportionate universalism giving emphasis to underprivileged sections of the population, within a population-wide strategy, to avoid broadening of health inequalities. In light of the above-mentioned limitations, further longitudinal studies incorporating survey data into the register data are recommended to estimate the magnitude of the problem by including adolescents with self-harm who are not seeking medical care. There is also a need for further studies to understand in depth the reasons why SEP affects girls more than boys. Finally future studies focusing on further investigating the relation between SEP and the different methods of self-harm, taking gender differences into consideration, are recommended.

## Conclusions

This study suggested that low parental SEP is associated with a higher risk of self-harm in adolescence, predominantly among girls. This association was not found among more severe cases of self-harm which may indicate that differences in health utilization between socioeconomic groups, showed in earlier studies, are less pronounced if adolescents suffer from self-harm with suicidal intention or substance-related disorders as underlying psychiatric diagnosis.

## Abbreviations

CI: confidence interval
HR: hazard ratio
DSM: diagnostic and statistical manual of mental disorders
ICD-10: international classification of diseases, 10th revision
LISA: longitudinal integration database for health insurance and labor market studies
NSSI: non suicidal self-harm
OR: odds ratio
SAS: statistical analysis system
SEP: socioeconomic position
SES: socioeconomic status
SII: self-inflicted injury
SYC: Stockholm Youth Cohort
WHO: World Health Organization
